# Gene and drug landing page aggregator

**DOI:** 10.1093/bioadv/vbac013

**Published:** 2022-02-28

**Authors:** Daniel J B Clarke, Maxim V Kuleshov, Zhuorui Xie, John E Evangelista, Marilyn R Meyers, Eryk Kropiwnicki, Sherry L Jenkins, Avi Ma’ayan

**Affiliations:** Department of Pharmacological Sciences, Mount Sinai Center for Bioinformatics, Icahn School of Medicine at Mount Sinai, New York, NY 10029, USA

## Abstract

**Motivation:**

Many biological and biomedical researchers commonly search for information about genes and drugs to gather knowledge from these resources. For the most part, such information is served as landing pages in disparate data repositories and web portals.

**Results:**

The Gene and Drug Landing Page Aggregator (GDLPA) provides users with access to 50 gene-centric and 19 drug-centric repositories, enabling them to retrieve landing pages corresponding to their gene and drug queries. Bringing these resources together into one dashboard that directs users to the landing pages across many resources can help centralize gene- and drug-centric knowledge, as well as raise awareness of available resources that may be missed when using standard search engines. To demonstrate the utility of GDLPA, case studies for the gene klotho and the drug remdesivir were developed. The first case study highlights the potential role of klotho as a drug target for aging and kidney disease, while the second study gathers knowledge regarding approval, usage, and safety for remdesivir, the first approved coronavirus disease 2019 therapeutic. Finally, based on our experience, we provide guidelines for developing effective landing pages for genes and drugs.

**Availability and implementation:**

GDLPA is open source and is available from: https://cfde-gene-pages.cloud/.

**Supplementary information:**

Supplementary data are available at *Bioinformatics Advances* online.

## 1 Introduction

Methods for experimentally collecting biomedical data are progressively becoming more efficient and less expensive leading to a rapid growth in data volume and diversity. For example, high-throughput methods for small molecule screening and generation of transcriptomics data have created an abundance of data related to genes and drugs which can be leveraged for reuse in bioinformatics analyses for hypothesis generation. Paralleling the growth of data generation, it is also increasingly less expensive to store data and make it publicly available via web-based databases and tools. For example, the Genotype-Tissue Expression (GTEx) program collects and serves omics data from human tissue samples to study gene expression and gene regulation in normal physiology; and these data are served for analysis and visualization on the GTEx web portal (GTEx Consortium, 2015). Among the analysis options offered by the GTEx portal are gene pages that include average expression of a gene in each tissue, and significant tissue specific expression quantitative trait loci (eQTLs). For an example of drug landing pages, DrugCentral provides drug-centric information about active ingredients, chemical entities, and pharmaceutical products with information about targets, mode of action, indications and pharmacological actions (Avram *et al.*, 2021). Data specific to single small molecule entities are available in single drug landing pages. These drug and gene page landing pages are just a few examples of disjointed resources that serve information about genes and drugs. Hence, it may be of benefit to biomedical researchers to have these resources accessible via one dashboard from which they can all be accessed. While most efforts have sought to aggregate metadata or data into a unified database for querying, this approach can become difficult to sustain. The Gene and Drug Landing Page Aggregator (GDLPA) is a dashboard that provides users with the ability to query gene and drug names to retrieve links to single gene and drug landing pages from 49 gene-centric (Supplementary Table S1A) and 19 drug-centric repositories (Supplementary Table S1B). Rather than storing and serving the information from these repositories in a new repository, we query these repositories on-demand and direct users to the information the repositories provide. The collection of repositories within GDLPA is federated, easy to maintain and expand, and remains continuously up to date.

## 2 Methods

GDLPA is a web-based application written in JavaScript and powered by NextJS, a React application framework for building server side rendered and incremental static regenerated (ISR) websites which share a single codebase for both the frontend and backend. GDLPA is compiled and bundled using the Node package manager and Docker making it cloud agnostic. Importantly, GDLPA has a manifest which is a codified listing of all the gene and drug resources. Such manifest includes descriptors, categorization tags, and callable functions for producing hyperlinks for a given gene or a drug query. The manifest is resolved at the query time based on the queried gene or drug, filtered based on the availability of the resulting hyperlink. The results are sorted based on a combination of whether the target site is tagged as primary or secondary source, and the prior clicks on cards representing each resource. Hyperlink availabilities are asserted by checking whether the target page returns a success status code (200). Incremental static regeneration (ISR) page caching is implemented to provide the benefits of static web pages including search engine optimization. However, we recompute the pages weekly to ensure that the information is kept current. In several cases, it is necessary to perform API requests to resolve gene identifiers with mygene.info (Wu *et al.*, 2014). These requests are memorized and made concurrently by implementing JavaScript Promises (Parker, 2015). The resulting manifest resolves dependencies as necessary, allowing each entry to remain independently defined while automatically deduplicating and caching requests. GDLPA provides users with gene–gene interactions (GGIs), drug–drug interactions (DDIs) and drug–gene interactions (DGIs) to make suggestions about related genes and drugs. GGIs from mRNA co-expression are computed from thousands of randomly selected RNA-seq samples processed uniformly with the ARCHS4 pipeline (Lachmann *et al.*, 2018). The top 10 genes are those with the highest Pearson correlation coefficient with the queried gene. GGIs from literature are computed from based on the most co-mentions genes with the queried gene in published abstracts based on a PubMed search (Lachmann *et al.*, 2019). DDIs from literature are computed from based on the most co-mentions drugs with the queried drug in published abstracts based on a PubMed search (Kropiwnicki *et al.*, 2021). DDIs based on the LINCS L1000 signatures are computed from the LINCS L1000 signatures (Wang *et al.*, 2016). For each drug, the strongest signature is selected and then signatures are compared with the cosine distance. Signatures are computed using the Characteristic Direction method (Clark *et al.*, 2014). DGIs from literature are computed from based on the most co-mentions drugs with the queried gene in published abstracts based on a PubMed search.

## 3 Results

**Fig. 1.**
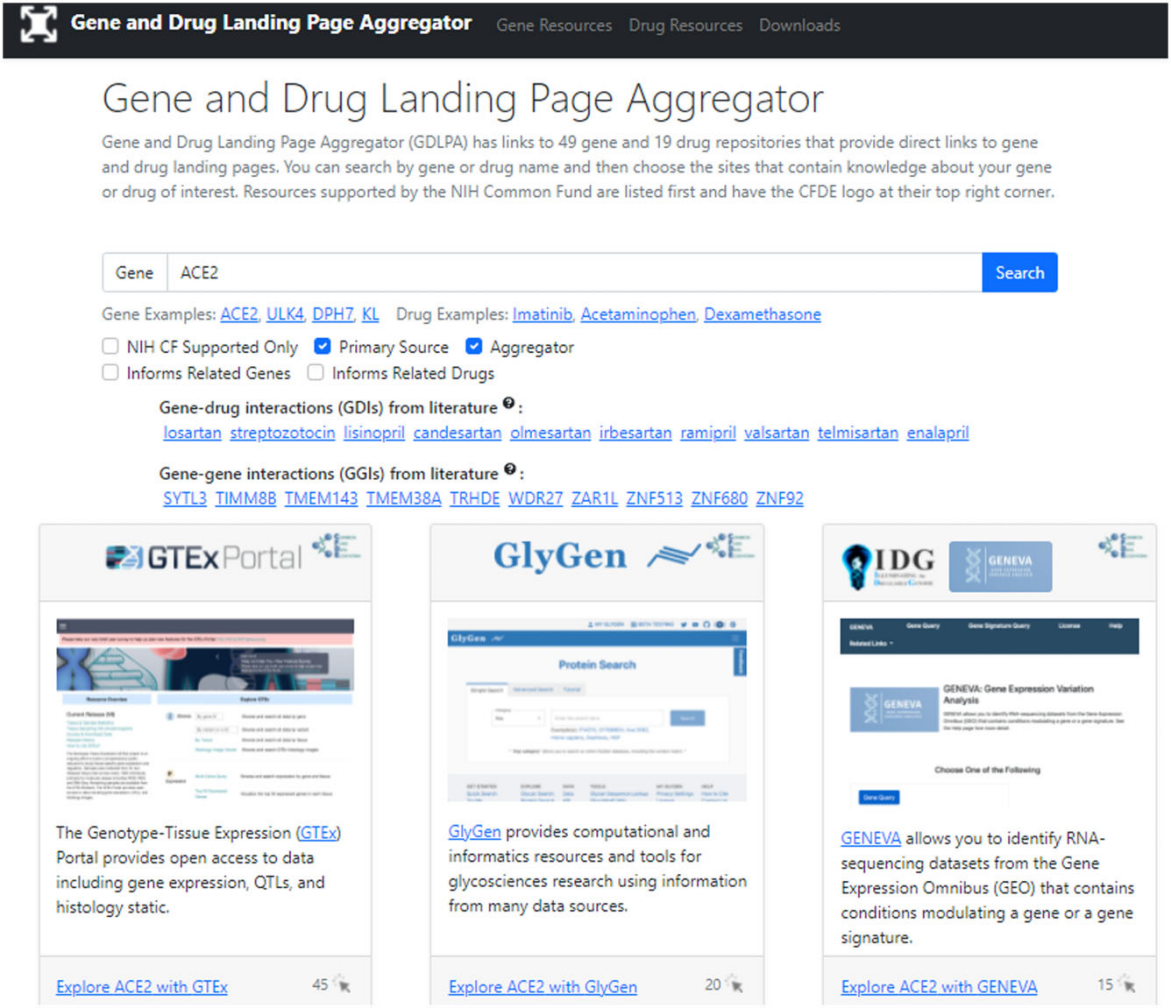
Screenshot from the user interface landing page of GDLPA with the example ACE2 gene as a query

**Fig. 2.**
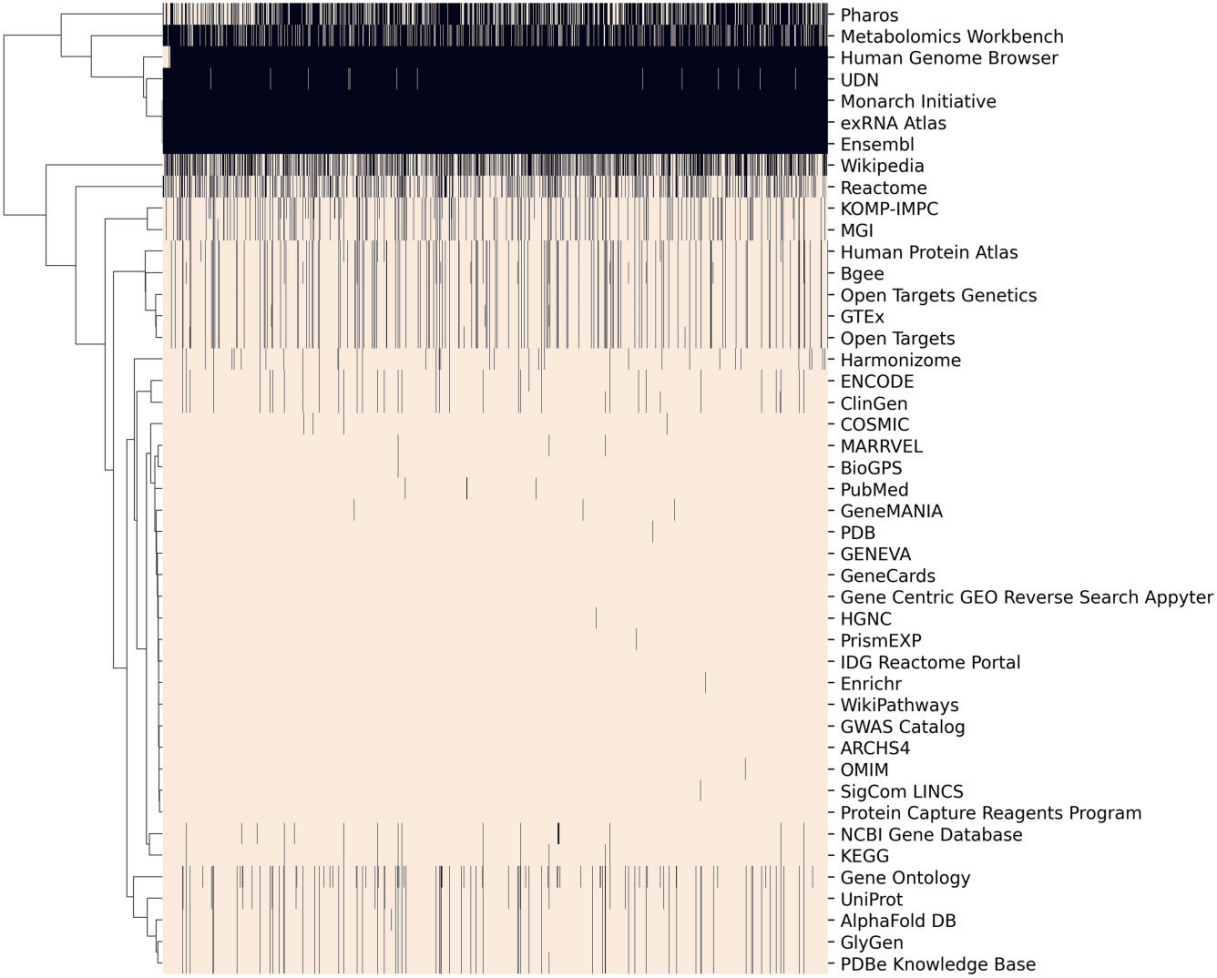
Availability of information about human genes by resource

**Fig. 3.**
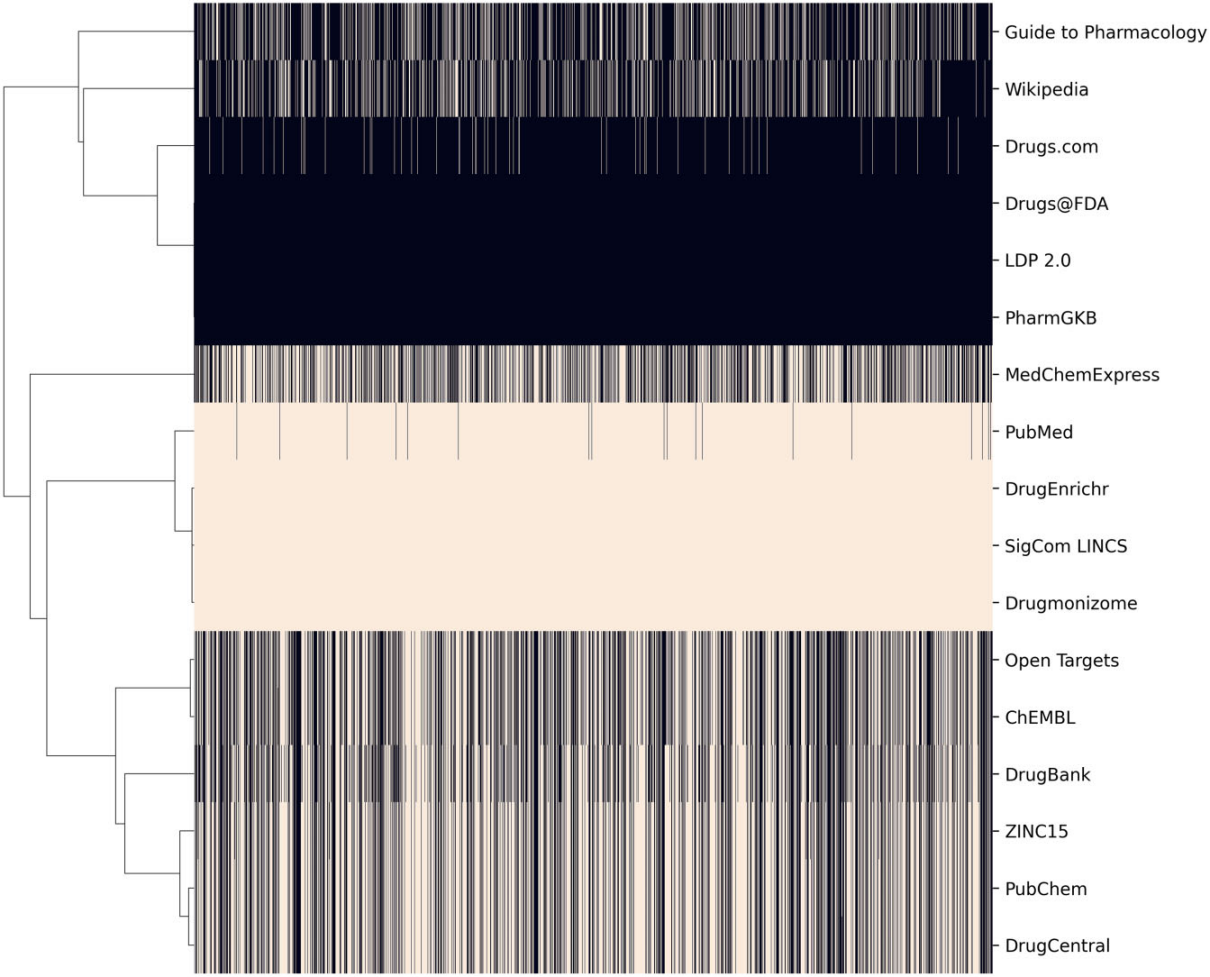
Availability of information about FDA approved drugs by resource. Beige color indicates a drug is represented in the resource.

The homepage of GDLPA includes a search bar for querying gene symbols and drug names. Filters near the search bar enable switching between gene and drug queries. Additionally, several toggleable options are provided for filtering the results by whether the resource is supported by the NIH Common, whether the resource is a primary source or an aggregator, and whether a gene resource has drug information or a drug resource contains gene information. After submitting a query, for example, the gene symbol ACE2, any resource that contains an ACE2 landing page will be represented on the search results as a card (Fig. 1). In addition, a list of other genes and drugs most related with the queried gene or drug based on mRNA co-expression and literature co-occurrence are generated at the top of the page. Clicking on any of these gene symbols or drug names leads to the submission of the gene or drug as a new query to GDLPA. The ‘Gene Resources’ and the ‘Drug Resources’ tabs include cards with descriptions and links to each of the available resources. The ‘Downloads’ tab includes links to download the manifests of gene and drug resources, as well as to matrices that list the availability of specific genes and drugs across each resource. These matrices were generated by querying all gene and drug terms from each resource. Such data coverage matrices are visualized as heatmaps for the gene resources (Fig. 2) and drug resources (Fig. 3). These coverage matrices will be updated periodically.

### 3.1 Case study 1: exploring knowledge about klotho

To demonstrate a use case using GDLPA, we searched for information about the gene klotho (KL), which is known to be downregulated in aging, highly expressed in kidney cells, and is important for healthy kidney function (Buchanan *et al.*, 2020). The search results from 52 gene-centric sources of complementary information about klotho provide collective knowledge about klotho that includes gene (NCBI Gene database, 49 720 nucleotides, Supplementary Fig. S1) and protein length (UniProt, 1012 amino acids, Supplementary Fig. S2), location on the chromosome (NCBI Gene database, 13q13.1, Supplementary Fig. S3), localization within the cell (OpenTargets, cell membrane and secreted, Supplementary Fig. S4), association with genetic diseases (GWAS Catalog, Type II Diabetes and diabetic nephropathy, Supplementary Fig. S5), the mechanisms by which KL expression is controlled by transcription factors (ARCHS4, FOXP1 and GBX2, Supplementary Fig. S6) and whether there are drugs and small molecules that may be used to induce the expression of klotho [RNA-seq-like Gene Centric Signature Reverse Search (RGCSRS) Appyter, staurosporine/up, Supplementary Fig. S7]. Interestingly, the coronavirus disease 2019 (COVID-19) Drug and Gene Set Library (Kuleshov *et al.*, 2020) in Enrichr (Kuleshov *et al.*, 2016) suggests that klotho is down-regulated by SARS-CoV-2 (Supplementary Fig. S8). This observation may explain the accelerated aging phenotype observed for some COVID-19 patients (Mongelli *et al.*, 2021). These are just a few exemplary knowledge parts about klotho out of many more available via the aggregated resources. Overall, this case study demonstrates how GDLPA can be used as a centralized site for biomedical researchers and clinicians to access a wide variety of information about a specific gene.

### 3.2 Case study 2: exploring knowledge about remdesivir

Users of GDLPA can also gather the most up-to-date information about specific drugs from various sources. This is particularly important when exploring accumulating knowledge about drugs that are actively undergoing investigation. The drug remdesivir is the first FDA-approved antiviral treatment for COVID-19, and new knowledge continues to be gathered about its effectiveness. We queried remdesivir with GDLPA, and this query illuminates a detailed timeline of its approval, usage, and safety. While DrugCentral (Avram *et al.*, 2021) only notes the date when remdesivir was granted full approval by the FDA (Supplementary Fig. S9), DrugBank (Wishart *et al.*, 2018) clarifies that an Emergency Use Authorization (EUA) was granted earlier, and that the indication was expanded after the full approval to treat non-hospitalized COVID-19 patients (Supplementary Fig. S10). Additionally, DrugBank lists the status of clinical trials involving remdesivir (Supplementary Fig. S11). In addition, PubChem (Kim *et al.*, 2021) exclusively provides full titles, registration information, dates and direct links to the detailed documentation of each clinical trial (Supplementary Fig. S12). Importantly, the OpenTargets resource provides information about the occurrence and frequency of adverse events experienced by patients that were prescribed remdesivir (Supplementary Fig. S13). By querying multiple sources with drug information, we can better understand the full context of the status of remdesivir as a COVID-19 treatment. GDLPA only requires one query and provides users with more complete and current information than each resource provides individually, which is critical for researchers to avoid relying on outdated knowledge or repeating experiments. Interestingly, none of the 19 drug resources supported by GDLPA provide detailed mechanisms of action of remdesivir in the context of COVID-19. It is expected that such knowledge will emerge in the coming months and years.

### 3.3 Guideline for developing gene and drug landing pages

GDLPA was created because we aimed to explore how gene-centric information is served by NIH Common Fund programs as part of our activities for the Common Fund Data Ecosystem (CFDE) program (Charbonneau *et al.*, 2021). While creating GDLPA, some resources were easier to integrate into the aggregator than others. Thus, we have developed some initial guidelines to biomedical portal developers that plan to host gene and drug landing pages. These guidelines are aligned with the findable, accessible, interoperable and reusable (FAIR) guiding principles (Wilkinson *et al.*, 2016). The information on the landing page should distinguish primary and secondary sources; as well as distinguish between data and metadata about the gene or drug. In the case of aggregating information from other sources, links and credit to those other sources should be provided. There should be a clear license that will guide the user on how the information provided on the landing page can be reused. In the case of aggregating information from other sources, links to the licenses from each of those external sources should be made available. The gene or drug identifiers should be mappable to established identifier systems such as HGNC, UniProt, Entrez Gene for genes and proteins, and PubChem, IUPAC, IUPHAR and SMILES for small molecules. The search engine that provides access to the landing page should be able to resolve synonymous identifiers. The gene or drug landing page should be accessible programmatically via API. The API should be well documented with a standard such as OpenAPI and deposited in an API repository such as SmartAPI. The gene symbols and drug names should be a part of the URL. The landing page should report a 404 HTTP status code if the symbol in the URL does not correspond to any available information. Landing pages should respond to HTTP HEAD requests and provide reliable information for optimal caching. The landing page should be persistent. If the landing page provides visualizations, these components should be embeddable in other sites. Users should be able to submit corrections and comments. Contact information about the content of the site should be provided. Experimental data vs. predictions should be made clear. Such guidelines will not only make it easier to add resources to GDLPA and make the site FAIRer, but these guidelines will also help resources become more useful. While GDLPA is simple, it can facilitate knowledge discovery by assisting biomedical and biological researchers to find knowledge about genes and drugs more easily.

## Software and data availability

The GDLPA web application is available at: https://cfde-gene-pages.cloud/.

GDLPA source code is available at: https://github.com/MaayanLab/cfde_gene_pages.

The GDLPA manifests for gene and drug landing pages information are available from: https://cfde-gene-pages.cloud/downloads.

## Supplementary Material

vbac013_Supplementary_Data
