# Supplementary material for: Gene and drug landing page aggregator
Source: Bioinform Adv. 2022 Feb 28;2(1):vbac013. doi: 10.1093/bioadv/vbac013 (PMC8969666; doi:10.1093/bioadv/vbac013)
Supplement: vbac013_Supplementary_Data [file vbac013_supplementary_data.pdf]

Supporting Online Materials for:

Gene and Drug Landing Page Aggregator

Daniel J. B. Clarke[1], Maxim V. Kuleshov[1], Zhuorui Xie[1], John E. Evangelista[1], Marilyn R. Meyers[1], Eryk Kropiwnicki[1], Sherry L. Jenkins[1], Avi Ma'ayan[1,*]

[1]Department of Pharmacological Sciences, Mount Sinai Center for Bioinformatics, Icahn School of Medicine at Mount Sinai, One Gustave L. Levy Place, Box 1603, New York, NY 10029, USA

*To whom correspondence should be addressed: avi.maayan@mssm.edu

Supporting Tables

Table S1A Resources that contain gene landing pages.

Resource name	URL	Aggregator	Primary Source	Common Fund	Ma'ayan Lab
AlphaFold DB	https://alphafold.ebi.ac.uk/		+		
ARCHS4	https://maayanlab.cloud/archs4/		+		+
Bgee	https://bgee.org/	+			
BioGPS	http://biogps.org		+		
ClinGen	https://www.clinicalgenome.org/		+		
COSMIC	https://cancer.sanger.ac.uk/cosmic		+		
ENCODE	https://www.encodeproject.org/		+		
Enrichr	https://maayanlab.cloud/Enrichr/	+			+
Ensembl	https://useast.ensembl.org/index.html		+		
exRNA Atlas	https://exrna-atlas.org/		+	+	
Gene Centric GEO Reverse Search Appyter	https://appyters.maayanlab.cloud/#Gene_Centric_GEO_Reverse_Search		+		+
Gene Ontology	http://geneontology.org/		+		
GeneCards	https://www.genecards.org/	+			
GeneMANIA	http://genemania.org	+			
GENEVA	https://genevatool.org/	+		+	
GlyGen	https://www.glygen.org/protein-search/		+	+	
GTEx	https://www.gtexportal.org/home/		+	+	
GWAS Catalog	https://www.ebi.ac.uk/gwas/home		+		

Harmonizome	https://maayanlab.cloud/Harmonizome/	+			+
HGNC	https://www.genenames.org/	+			
Human Genome Browser	https://genome.ucsc.edu/		+		
Human Protein Atlas	https://www.proteinatlas.org		+		
IDG Reactome Portal	https://idg.reactome.org/		+	+	
KEGG	https://www.genome.jp/kegg/		+		
KOMP-IMPC	https://www.mousephenotype.org		+	+	
MARRVEL	http://marrvel.org/	+			
Metabolomics Workbench	https://www.metabolomicsworkbench.org/		+	+	
MGI	http://www.informatics.jax.org/		+		
Monarch Initiative	https://monarchinitiative.org/		+		
NCBI Gene Database	https://www.ncbi.nlm.nih.gov/gene/	+			
OMIM	https://omim.org/		+		
Open Targets	https://platform.opentargets.org/	+			
Open Targets Genetics	https://genetics.opentargets.org/	+			
PDB	https://www.rcsb.org/		+		
PDBe Knowledge Base	https://www.ebi.ac.uk/pdbe/pdbe-kb		+		
Pharos	https://pharos.nih.gov/	+		+	
PrismEXP	https://appyters.maayanlab.cloud/#PrismEXP		+		+
Protein Capture Reagents Program	https://proteincapture.org/	+		+	
PubMed	https://pubmed.ncbi.nlm.nih.gov/	+			
Reactome	https://reactome.org/		+	+	
RGCSRS	https://appyters.maayanlab.cloud/#/L1000_RNAseq_Gene_Search		+		+
SigCom LINCS	https://maayanlab.cloud/sigcom-lincs	+		+	+
STITCH	http://stitch.embl.de/	+			
STRING	https://string-db.org/	+			
Therapeutic Target Database	http://db.idrblab.net/ttd/	+			
UDN	https://undiagnosed.hms.harvard.edu/genes/		+	+	
UniProt	https://www.uniprot.org/	+			
WikiPathways	https://www.wikipathways.org/	+			
Wikipedia	https://en.wikipedia.org/	+			

Table S1B Resources that contain drug landing pages

Resource name	URL	Aggregator	Primary Source	Common Fund	Ma'ayan Lab
ChEMBL	https://www.ebi.ac.uk/chembl/	+			
DrugBank	https://go.drugbank.com/	+			
DrugCentral	https://drugcentral.org/	+		+	
DrugEnrichr	https://maayanlab.cloud/DrugEnrichr/	+			+
Drugmonizome	https://maayanlab.cloud/drugmonizome/	+		+	+
Drugs.com	https://www.drugs.com/	+			
Drugs@FDA	https://www.accessdata.fda.gov/scripts/cder/daf/		+		
Guide to Pharmacology	https://www.guidetopharmacology.org/	+			
LDP 2.0	http://lincsportal.ccs.miami.edu/signatures/home		+	+	
MedChemExpress	https://www.medchemexpress.com/	+			
Open Targets	https://platform.opentargets.org/	+			
PharmGKB	https://www.pharmgkb.org/	+			
PubChem	https://pubchem.ncbi.nlm.nih.gov/	+			
PubMed	https://pubmed.ncbi.nlm.nih.gov/	+			
SigCom LINCS	https://maayanlab.cloud/sigcom-lincs	+		+	+
STITCH	http://stitch.embl.de/	+			
Theraperutic Target Database	http://db.idrblab.net/ttd/	+			
Wikipedia	https://en.wikipedia.org/	+			
ZINC15	https://zinc15.docking.org/		+	+	

Supporting Figures

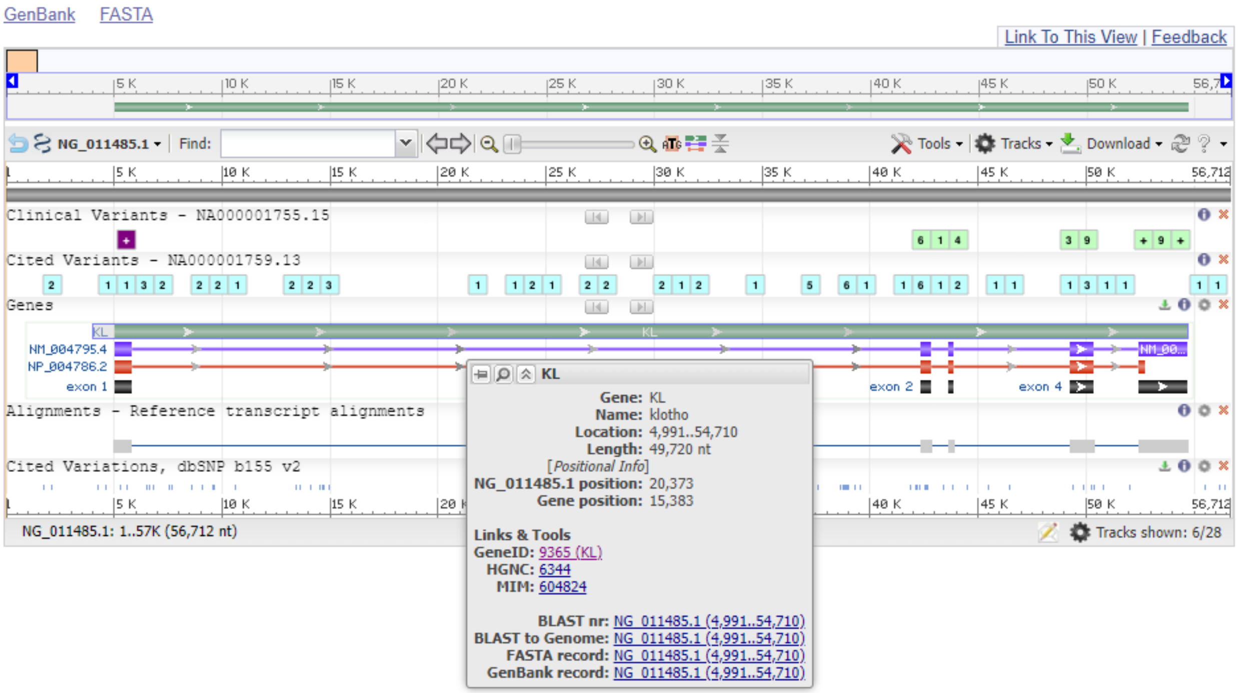

Fig. S1 Gene size information from the NCBI Gene database.

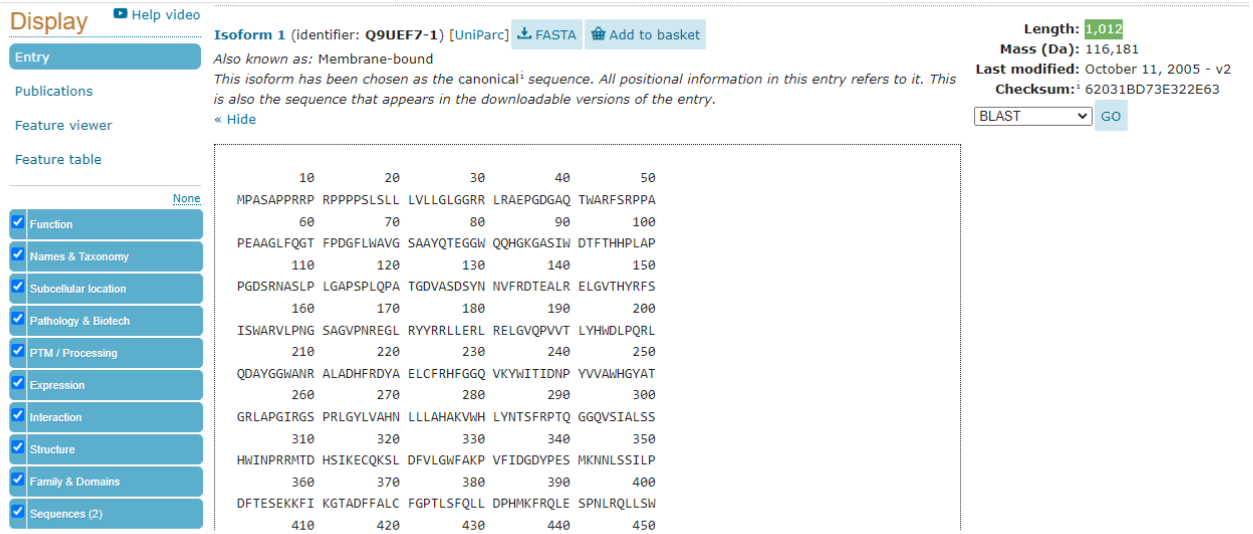

Fig. S2 Protein size information from the UniProt database.

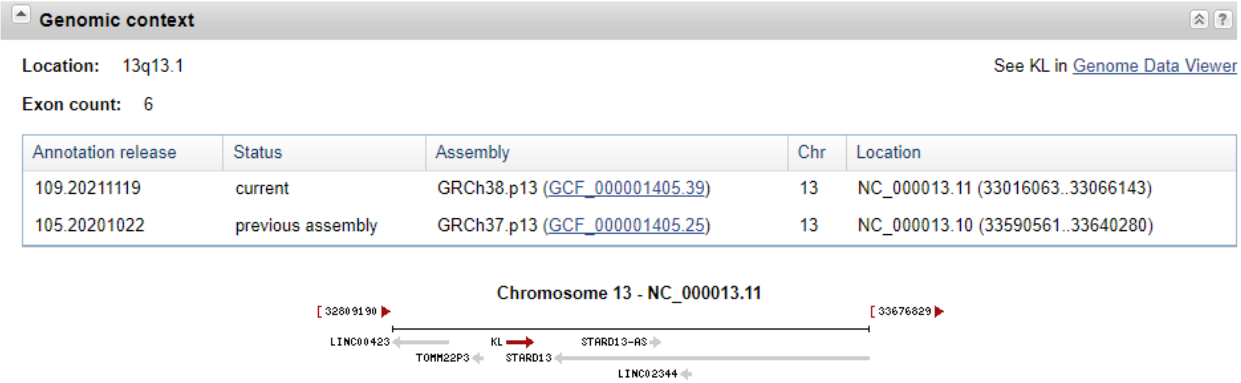

Annotation release	Status	Assembly	Chr	Location
109.20211119	current	GRCh38.p13 (GCF_000001405.39)	13	NC_000013.11 (33016063..33066143)
105.20201022	previous assembly	GRCh37.p13 (GCF_000001405.25)	13	NC_000013.10 (33590561..33640280)

Fig. S3 Gene location information from the NCBI Gene database.

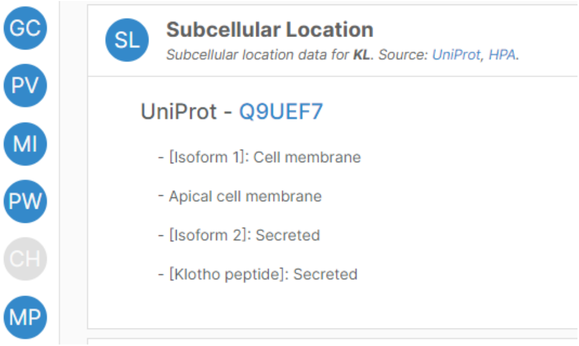

Fig. S4 Gene subcellular location information from OpenTargets.

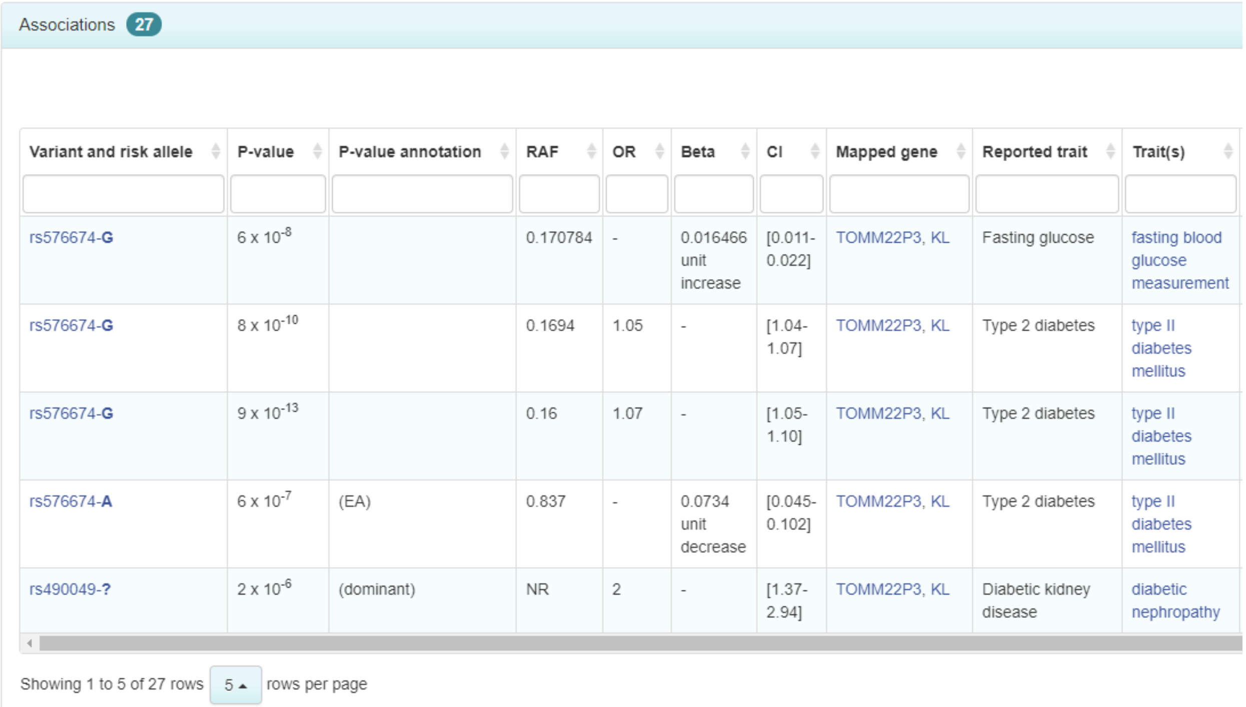

Variant and risk allele	P-value	P-value annotation	RAF	OR	Beta	CI	Mapped gene	Reported trait	Trait(s)
rs576674-G	6×10^{-8}		0.170784	-	0.016466 unit increase	[0.011-0.022]	TOMM22P3, KL	Fasting glucose	fasting blood glucose measurement
rs576674-G	8×10^{-10}		0.1694	1.05	-	[1.04-1.07]	TOMM22P3, KL	Type 2 diabetes	type II diabetes mellitus
rs576674-G	9×10^{-13}		0.16	1.07	-	[1.05-1.10]	TOMM22P3, KL	Type 2 diabetes	type II diabetes mellitus
rs576674-A	6×10^{-7}	(EA)	0.837	-	0.0734 unit decrease	[0.045-0.102]	TOMM22P3, KL	Type 2 diabetes	type II diabetes mellitus
rs490049-?	2×10^{-6}	(dominant)	NR	2	-	[1.37-2.94]	TOMM22P3, KL	Diabetic kidney disease	diabetic nephropathy

Fig. S5 Gene-disease associations from GWAS Catalog.

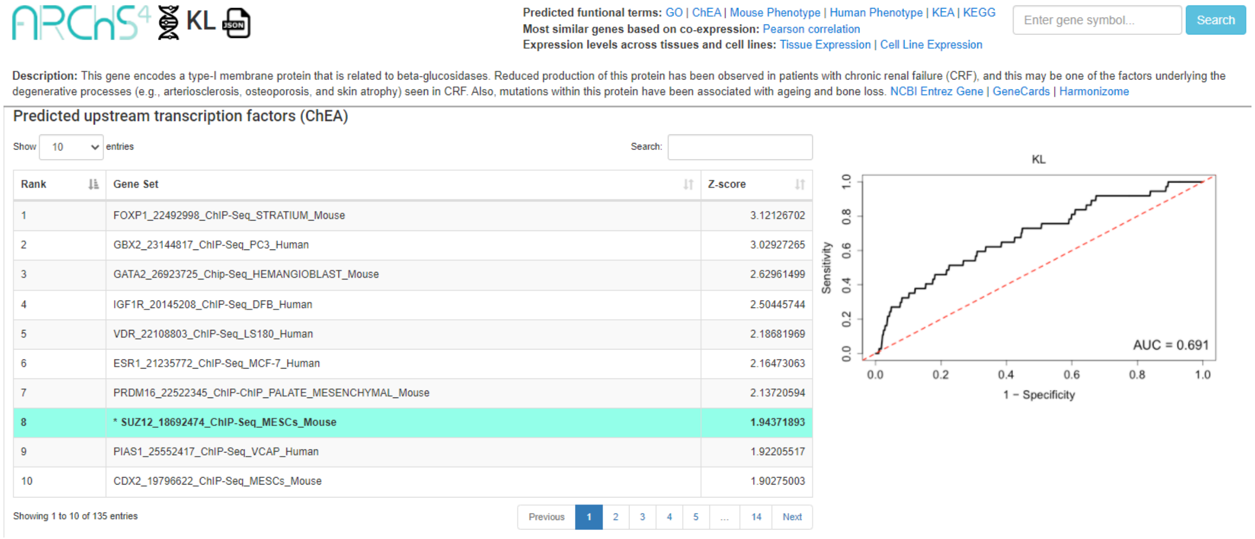

Fig. S6 Predicted transcription factor regulators from ARCHS4 gene pages.

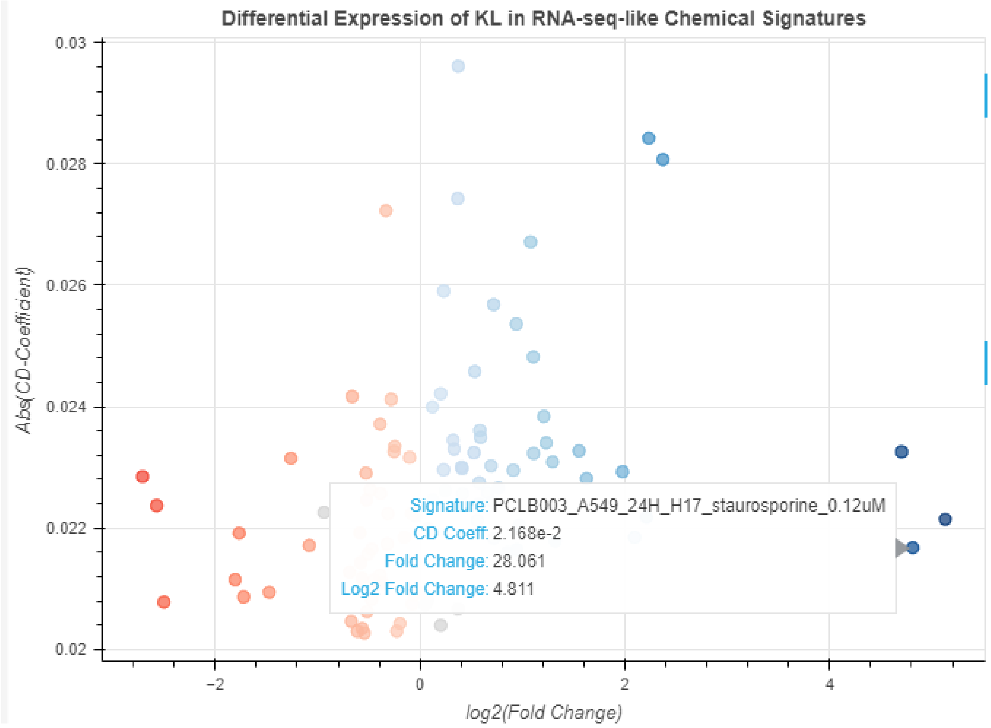

Fig. S7 Small molecules predicted to maximally up- or down-regulate the expression of KL based on the L1000 data and the RNA-seq-like Gene Centric Signature Reverse Search (RGCSRS) Appyter.

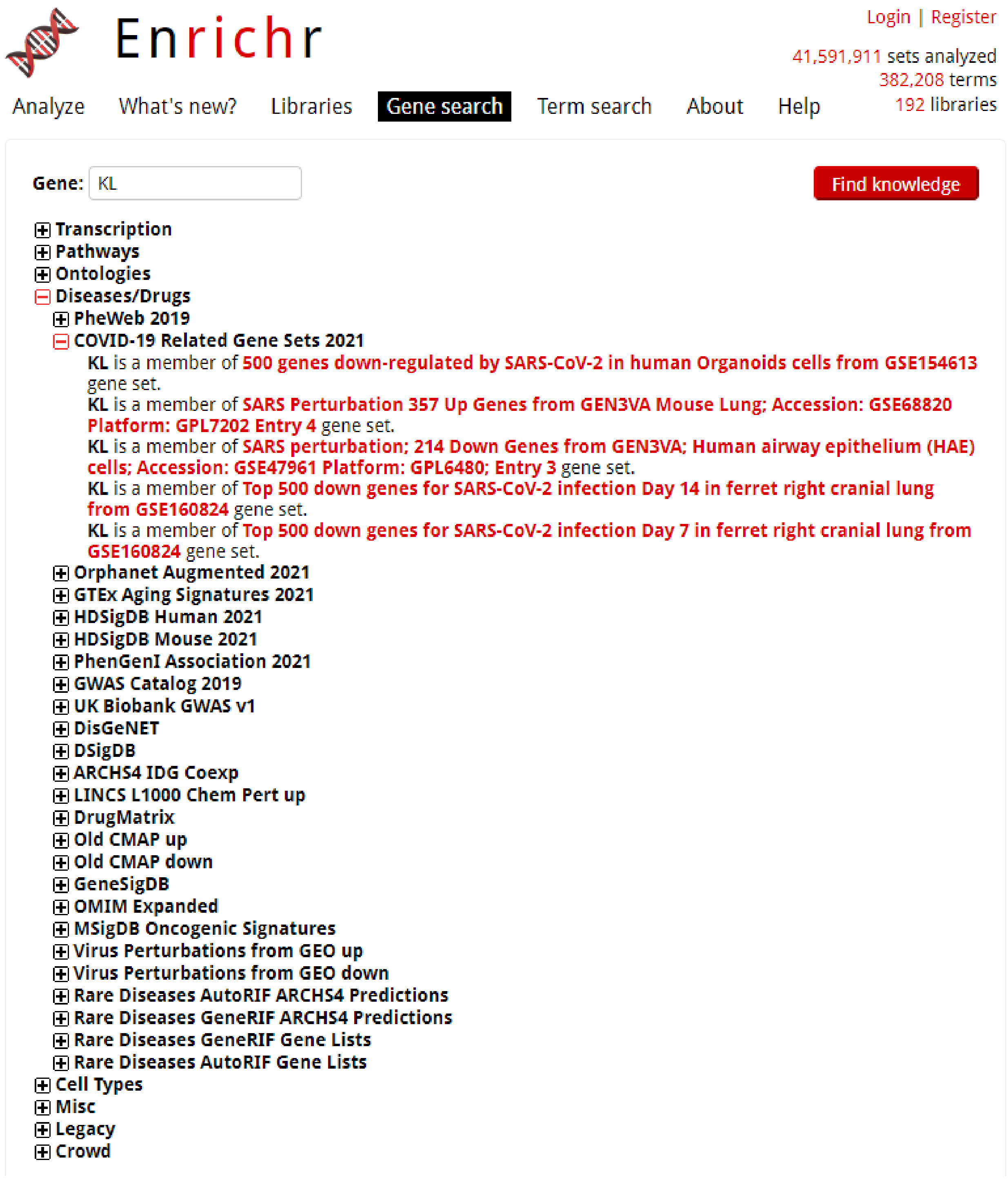

Fig. S8 KL presence in gene set related to SARS-CoV-2 from the Enrichr gene set library.

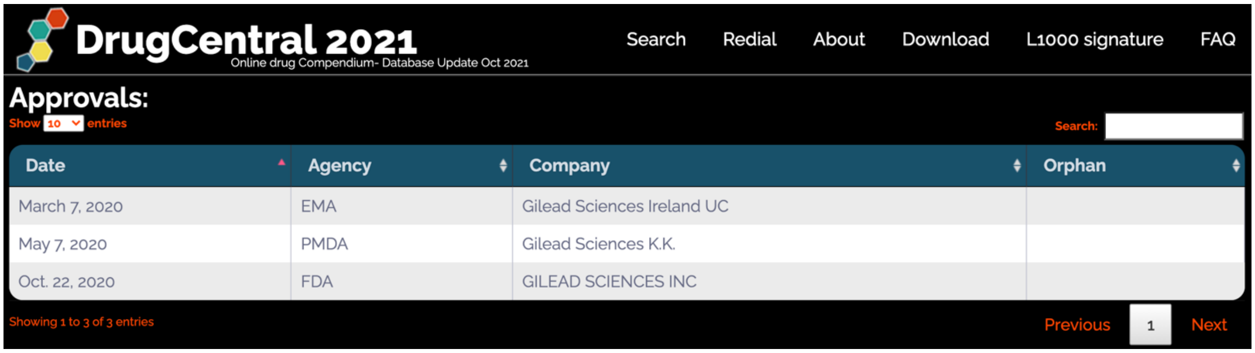

DrugCentral 2021
Online drug Compendium- Database Update Oct 2021
Search Redial About Download L1000 signature FAQ

Approvals:
Show 10 entries
Search:

Date	Agency	Company	Orphan
March 7, 2020	EMA	Gilead Sciences Ireland UC	
May 7, 2020	PMDA	Gilead Sciences K.K.	
Oct. 22, 2020	FDA	GILEAD SCIENCES INC	

Showing 1 to 3 of 3 entries
Previous 1 Next

Fig. S9 Table from DrugCentral listing remdesivir approvals by date, approving agency, and manufacturing company.

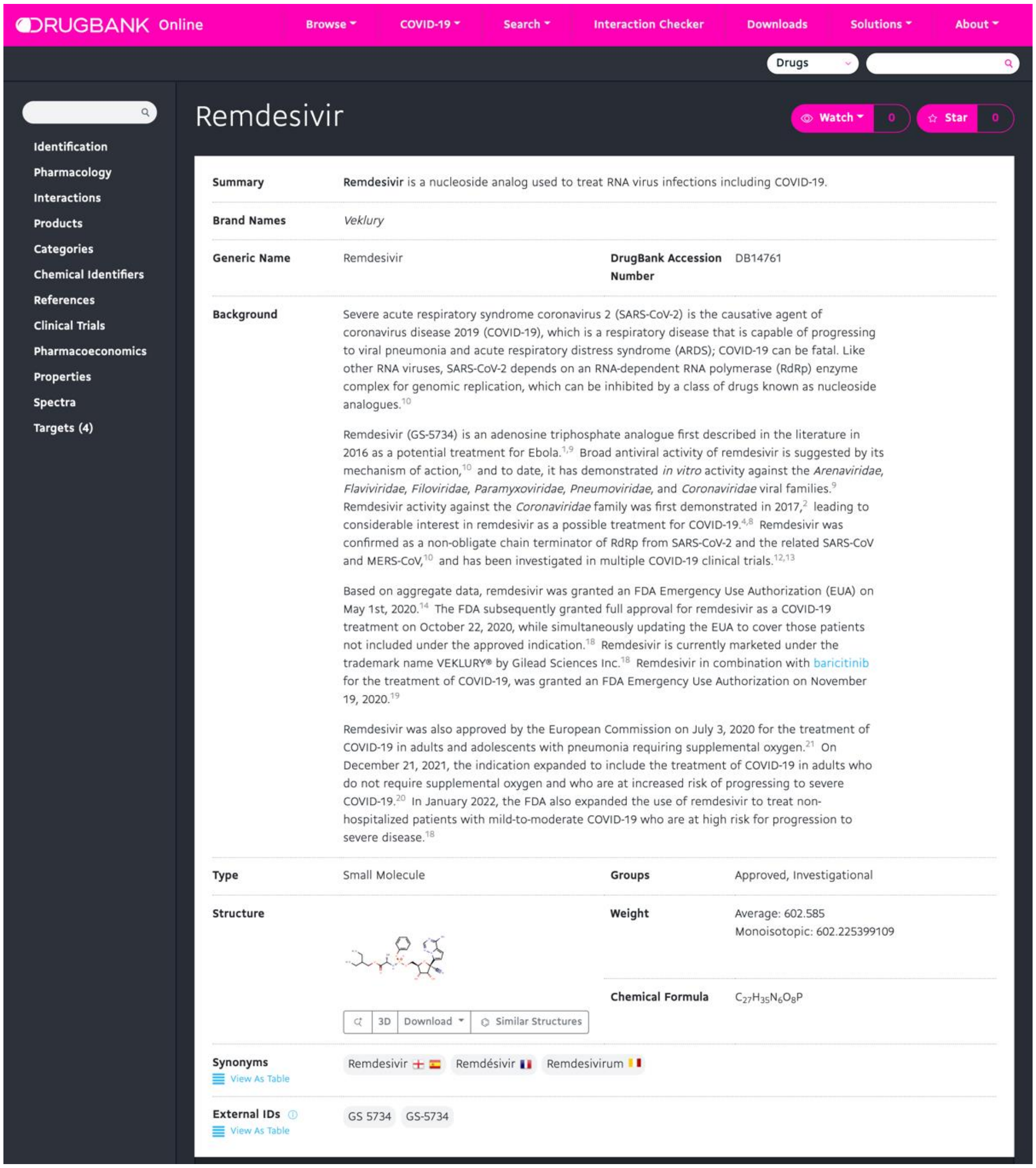

DRUGBANK Online
Browse COVID-19 Search Interaction Checker Downloads Solutions About
Drugs

Identification
Pharmacology
Interactions
Products
Categories
Chemical Identifiers
References
Clinical Trials
Pharmacoeconomics
Properties
Spectra
Targets (4)

Remdesivir
Watch 0 Star 0

Summary **Remdesivir** is a nucleoside analog used to treat RNA virus infections including COVID-19.

Brand Names *Veklury*

Generic Name Remdesivir **DrugBank Accession Number** DB14761

Background Severe acute respiratory syndrome coronavirus 2 (SARS-CoV-2) is the causative agent of coronavirus disease 2019 (COVID-19), which is a respiratory disease that is capable of progressing to viral pneumonia and acute respiratory distress syndrome (ARDS); COVID-19 can be fatal. Like other RNA viruses, SARS-CoV-2 depends on an RNA-dependent RNA polymerase (RdRp) enzyme complex for genomic replication, which can be inhibited by a class of drugs known as nucleoside analogues.[10]

Remdesivir (GS-5734) is an adenosine triphosphate analogue first described in the literature in 2016 as a potential treatment for Ebola.[1,9] Broad antiviral activity of remdesivir is suggested by its mechanism of action,[10] and to date, it has demonstrated *in vitro* activity against the *Arenaviridae*, *Flaviviridae*, *Filoviridae*, *Paramyxoviridae*, *Pneumoviridae*, and *Coronaviridae* viral families.[9] Remdesivir activity against the *Coronaviridae* family was first demonstrated in 2017,[2] leading to considerable interest in remdesivir as a possible treatment for COVID-19.[4,8] Remdesivir was confirmed as a non-obligate chain terminator of RdRp from SARS-CoV-2 and the related SARS-CoV and MERS-CoV,[10] and has been investigated in multiple COVID-19 clinical trials.[12,13]

Based on aggregate data, remdesivir was granted an FDA Emergency Use Authorization (EUA) on May 1st, 2020.[14] The FDA subsequently granted full approval for remdesivir as a COVID-19 treatment on October 22, 2020, while simultaneously updating the EUA to cover those patients not included under the approved indication.[18] Remdesivir is currently marketed under the trademark name VEKLURY® by Gilead Sciences Inc.[18] Remdesivir in combination with baricitinib for the treatment of COVID-19, was granted an FDA Emergency Use Authorization on November 19, 2020.[19]

Remdesivir was also approved by the European Commission on July 3, 2020 for the treatment of COVID-19 in adults and adolescents with pneumonia requiring supplemental oxygen.[21] On December 21, 2021, the indication expanded to include the treatment of COVID-19 in adults who do not require supplemental oxygen and who are at increased risk of progressing to severe COVID-19.[20] In January 2022, the FDA also expanded the use of remdesivir to treat non-hospitalized patients with mild-to-moderate COVID-19 who are at high risk for progression to severe disease.[18]

Type Small Molecule **Groups** Approved, Investigational

Structure **Weight** Average: 602.585 Monoisotopic: 602.225399109

Chemical Formula $C_{27}H_{35}N_6O_8P$

3D Download Similar Structures

Synonyms View As Table — Remdesivir Remdésivir Remdesivirum

External IDs View As Table — GS 5734 GS-5734

Fig. S10 Background information on remdesivir from DrugBank, including timeline of Emergency Use Authorizations, full approvals, and expansions of indication.

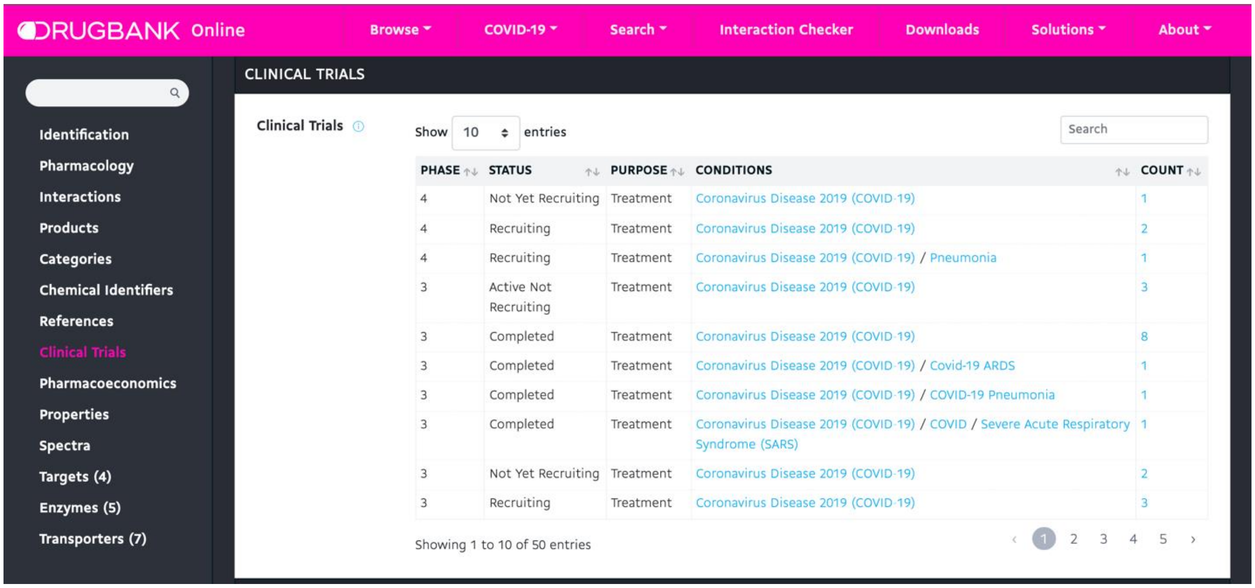

Fig. S11 List of clinical trials including remdesivir on DrugBank by phase, status, purpose, and the condition involved.

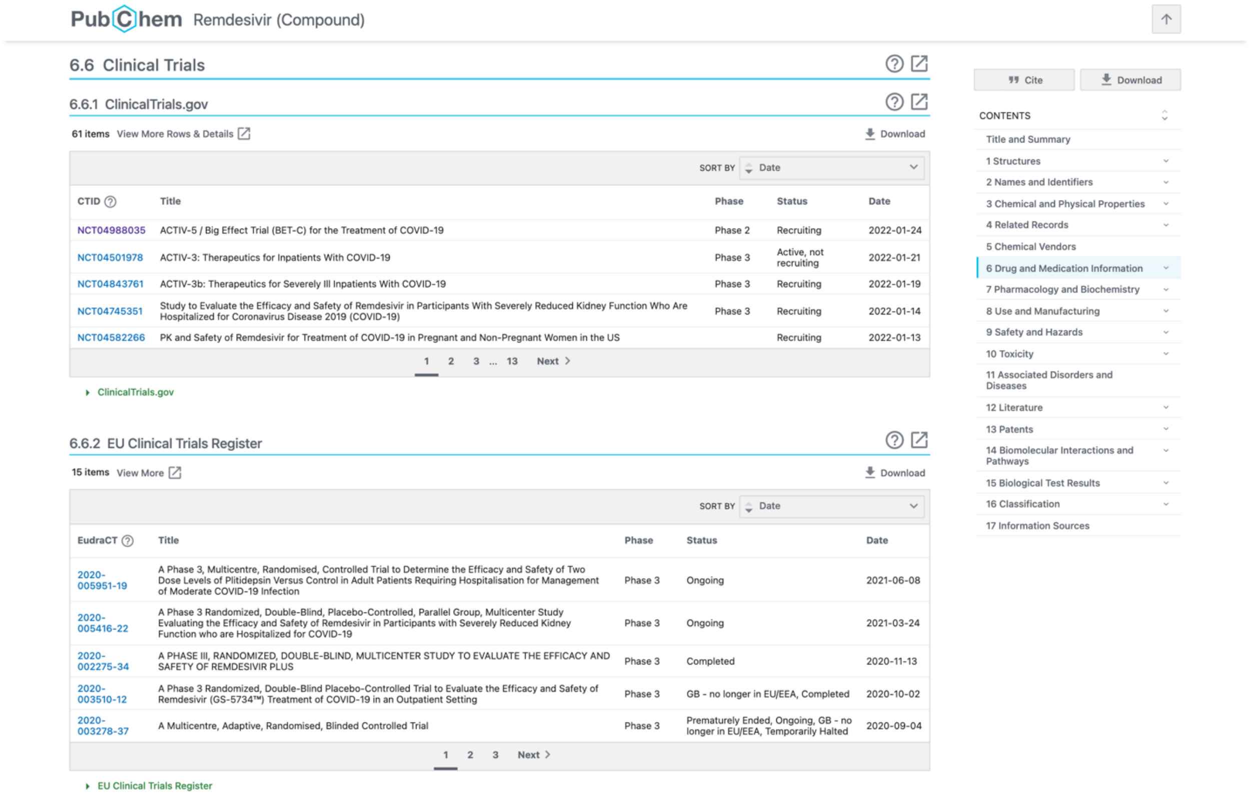

Fig. S12 PubChem tables of all clinical trials involving remdesivir, organized by registration agency and listed by identifier, title, phase, status, and date.

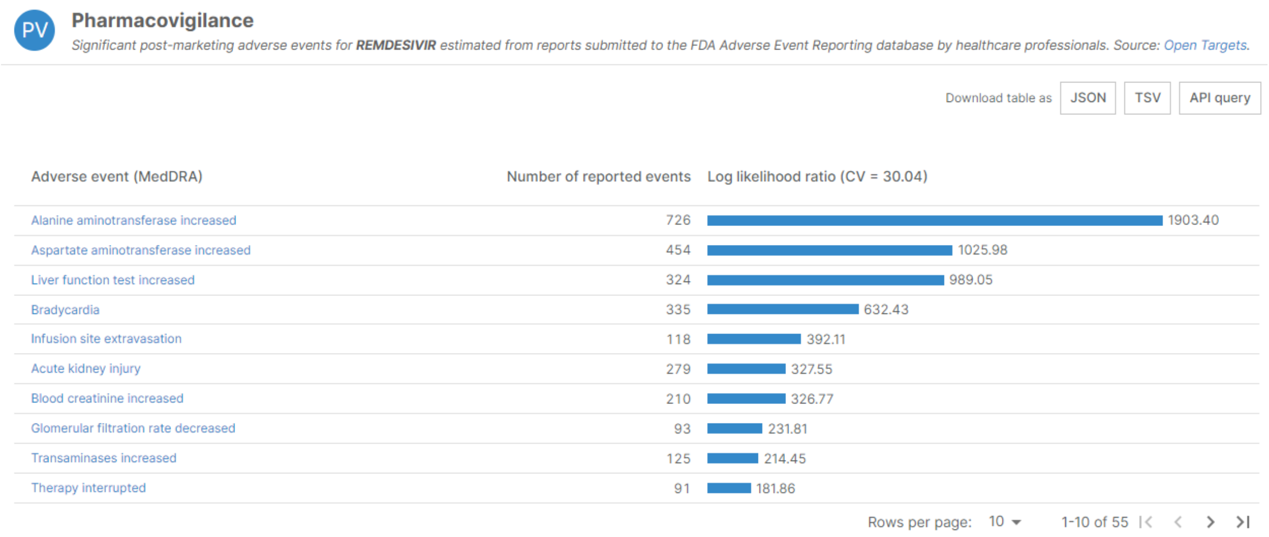

PV **Pharmacovigilance**

Significant post-marketing adverse events for ***REMDESIVIR*** *estimated from reports submitted to the FDA Adverse Event Reporting database by healthcare professionals. Source: Open Targets.*

Download table as JSON TSV API query

Adverse event (MedDRA)	Number of reported events	Log likelihood ratio (CV = 30.04)
Alanine aminotransferase increased	726	1903.40
Aspartate aminotransferase increased	454	1025.98
Liver function test increased	324	989.05
Bradycardia	335	632.43
Infusion site extravasation	118	392.11
Acute kidney injury	279	327.55
Blood creatinine increased	210	326.77
Glomerular filtration rate decreased	93	231.81
Transaminases increased	125	214.45
Therapy interrupted	91	181.86

Rows per page: 10 1-10 of 55

Fig. S13 Pharmacovigilance information about remdesivir from OpenTargets.
